# Intracerebral immune complex formation induces inflammation in the brain that depends on Fc receptor interaction

**DOI:** 10.1007/s00401-012-0995-3

**Published:** 2012-05-18

**Authors:** Jessica L. Teeling, Roxana O. Carare, Martin J. Glennie, V. Hugh Perry

**Affiliations:** 1 Centre for of Biological Sciences, University of Southampton, Southampton General Hospital, Mail Point 840, South Lab and Path Block, Southampton, UK; 2 Clinical Neurosciences, Faculty of Medicine, University of Southampton, Southampton, UK; 3 Cancer Sciences, Faculty of Medicine, University of Southampton, Southampton, UK

**Keywords:** Fc receptor, Antibody, Immunotherapy, Microglial activation

## Abstract

In this study, we investigate the underlying mechanisms of antibody-mediated inflammation in the brain. We show that immune complexes formed in the brain parenchyma generate a robust and long-lasting inflammatory response, characterized by increased expression of the microglia markers CD11b, CD68 and FcRII/III, but no neutrophil recruitment. In addition to these histological changes, we observed transient behavioural changes that coincided with the inflammatory response in the brain. The inflammatory and behavioural changes were absent in Fc-gamma chain (Fcγ)-deficient mice, while C1q-deficient mice were not different from wild-type mice. We conclude that, in the presence of antigen, antibodies can lead to a local immune complex-mediated inflammatory reaction in the brain parenchyma and indirectly induce neuronal tissue damage through recruitment and activation of microglia via Fcγ receptors. These observations may have important implications for the development of therapeutic antibodies directed against neuronal antigens used for therapeutic intervention in neurological diseases.

## Introduction

An inevitable consequence of an ageing population is an increased incidence of neurodegeneratative diseases, such as Alzheimer’s disease, Parkinson’s disease and age-related macular degeneration. Recent experimental and clinical studies have provided evidence for both innate and adaptive immune activation in the pathogenesis of these debilitating disorders. For example, microglia activation is typically associated with any neuropathology and there is increasing evidence that (auto)-antibodies against brain-reactive antigens are associated with clinical symptoms [11, 28]. Genome wide association studies (GWAS) in AD [21] and AMD [35] provide further evidence for the involvement of the immune system in disease pathogenesis. The young and healthy CNS is an immune privileged site where immune surveillance and inflammation is tightly regulated [4, 15]. The presence of an intact blood–brain barrier (BBB) combined with the unique microenvironment of the brain, ensures that the reactions to an inflammatory challenge, such as endotoxin (LPS) or cytokines, are attenuated [30]. We and others have shown that the healthy CNS parenchyma is able to modify leucocyte responses to acute injury [2, 3], but it is less clear if, and how, the brain controls antibody-mediated responses, and whether these responses are altered under neuroinflammatory conditions or age-related pathology. Antibodies may mediate tissue damage when they form immune complexes and recruit cytotoxic effector cells, such as macrophages via their Fcγ receptors (FcγRs) or by activating complement [34]. The interaction with FcγRs stimulates cell signalling in the effector cell that ultimately results in phagocytosis and/or release of inflammatory or cytotoxic mediators. These responses are well described in peripheral tissues, using the (reversed) Arthus reaction, a well accepted experimental model of antibody-mediated inflammation [10]. In the presence of an intact BBB, IgG is only present in the healthy brain at very low levels relative to plasma levels [32] and the effector cells, such as microglia and perivascular macrophages, express detectable but low levels of FcγRs [31]. However, expression of FcγRs is enhanced on microglia following treatment with IFN-γ, TNF-α and LPS in vitro [26], after intracerebral injection of LPS [17] and, as we have recently shown, during experimental chronic neurodegeneration [27]. Despite these observations we have limited knowledge of the consequences of immune complex formation in the CNS, the associated inflammatory response and the function of the different FcγRs in the CNS. The growing incidence of neurodegenerative conditions in the human population, and the interest in the use of antibody-based immunotherapy to treat these diseases, highlights the need to understand the possible consequences of antibody-mediated inflammation in the CNS parenchyma.

Davidoff et al. [12] were the first to describe antibody-mediated inflammation in the brain and showed that the Arthus reaction in the rabbit brain resembled that seen in the skin and other peripheral tissues. However, it is likely that the relatively crude techniques used in this study significantly disturbed the unique vasculature and microenvironment of the brain making it difficult to interpret the results. More recently, Lister and Hickey [25] reported that immune complexes can be formed in the microvasculature of the brain, resulting in complement activation, increased microvascular permeability and leucocyte adhesion. However, this study was restricted to the role of immune complexes in the meningeal compartment and not in the brain parenchyma.

The aim of the current study was to investigate the acute and long-term consequences of immune complex formation in the brain parenchyma, using a model antigen widely used in the study of peripheral antibody-mediated responses. We show that immune complex formation in the brain parenchyma results in neuroinflammatory and behavioural changes that depend on FcγR interactions. Apart from further understanding of antibody-mediated responses in the brain in general, our study provides insight into the complications reported following anti-Aβ immunization, such as micro-haemorrhages and increased cerebral amyloid angiopathy (CAA) [6, 46, 47].

## Materials and methods

### Mice

BALB/c mice were obtained from Charles River (Margate, UK) and bred and maintained in local facilities. Fcγ chain deficient (Fcγ−/−) originally described by Takai et al. [42] were obtained from The Jackson Laboratory and back crossed onto a BALB/c background. C1q-deficient (C1q−/−mice were obtained from Dr Aras Kadioglu (Leicester, UK) with permission from Professor Marina Botto (London, UK) [7]. Animal experiments were carried out with approval from the local Committee for Ethics at the University of Southampton and were performed under a Home Office license.

### Immunization

8 week old BALB/c mice were immunized against ovalbumin (OVA) by intraperitoneal injection of 50 μg OVA (Sigma) in the presence of Alum (1:1 ratio, Alum imject, Pierce). Mice were boosted three times (2, 4 and 6 weeks) by intraperitoneal injection of 100 μg OVA in saline. Three days after the last OVA injection, OVA was microinjected into the striatum.

### Immune complex formation

Immune complex-mediated inflammation was initiated by a cerebral injection of OVA in OVA-immunized or non-immunized control animals. Mice were anaesthetized by intraperitoneal injection of 0.1 ml/5 g body weight Avertin (2,2,2 tribromoethanol in tertiary amyl alcohol) and placed in a stereotaxic frame (Kopf Instruments, Tujunga, CA, USA). OVA (10 μg in 1 μl) was injected into the striatum (bregma +1 mm anterior, lateral +1.5 mm, 2.5 mm deep) by a minimally invasive technique using a fine glass micropipette with a diameter of <50 μm (Sigma). Tissue was collected after 24 h, 3 days, 7 days, 14 days and 28 days. These time points were chosen based on the kinetics of immune complex-mediated inflammation in peripheral organs [40] or microglial activation in the brain parenchyma following LPS challenge [17]. Under terminal anaesthesia, blood samples were collected by cardiac puncture, and after transcardial perfusion using heparinized saline, brains and spleen were removed and snap frozen in OCT embedding medium. For immunohistological examination and quantification studies, brains were sectioned in a coronal plane on a cryostat (Leica 17–20).

### Immunocytochemistry

Serial sections of brain, 10 μm thick, were air-dried and fixed in cold ethanol for 10 min at 4 °C, and stained for the presence of immune complexes. Rabbit anti-OVA (Sigma, UK) was used to detect the antigen OVA and complement activation was identified using antibodies against C3 (FITC conjugated rabbit anti-C3, Cappel). Mouse IgG was identified using FITC labelled F(ab′)_2_ fragments of goat anti-mouse IgG (Sigma, UK). Phenotypic changes in macrophages were assessed using rat anti-mouse F4/80 (serotec), CD11b (5C6, Serotec), CD68 (FA11, Serotec), MHC class II (Bioscience) and CD16/CD32 (FCR4G8, FcRII/III, Serotec). The presence of neutrophils was assessed with MBS-1, an in-house produced polyclonal antibody, generated as described elsewhere [43]. The presence of platelets was assessed with a rat anti-mouse gpII1/IIIb mAb (CD41, Serotec) and T cells using rat anti-mouse CD3 (KT3, Serotec). Biotinylated secondary antibodies and HRP-conjugated streptavidin were from Vector (UK) and the chromogen substrate DAB from Sigma (UK). Alexa Fluor 488 or Alexa Fluor 546 conjugated secondary antibodies were obtained from Invitrogen (Molecular Probes, Oregon, USA). Mounted sections were cover-slipped using Vectashield (Hard set, with DAPI, Vector, UK). The intensity of the macrophage markers CD11b, F4/80 and MHC class II was quantified using the Leica analysis software on a Leica microscope. The expression level of macrophage markers was quantified by taking four images at 10× magnification per injected hemisphere (field). The total number of pixels of DAB positive staining per field was recorded. Data was analysed by One-way ANOVA followed by Dunnett’s post hoc test. *p* < 0.05 was considered significantly different.

### OVA antibody ELISA

Sera from OVA-immunized mice was serially diluted onto OVA coated plates (10 μg/ml in PBS; maxiSorb, Nunc) followed by incubation with biotinylated horse-anti-mouse IgG (Vector, UK) for determination of total OVA-specific IgG levels. Subclasses were determined by IgG1- and IgG2a-specific antibodies (Serotec, UK). Binding of OVA-specific antibodies was detected by poly-streptavidin (Sanquin, The Netherlands) and visualized using TMB/H_2_0_2_ substrate (R&D systems, UK).

### Assessment of behaviour

Circling behaviour was carried out in an opaque cylindrical bowl of 10 cm circumference, with a clearly labelled mid-point that was used as a reference as to how many times the mouse crosses the line and in which direction. Left and right turns were counted when the tip of the nose crossed this reference line, over a 1-min period. The left and right turning behaviour was measured at day −1 for baseline measurements and at day 1, 3, 7 and 14 after intracerebral OVA injection.

### Statistical analysis

Quantification of immune activation markers and behavioural data was analysed by one-way analysis of variance (ANOVA) followed, if significant, by Dunnett’s post-test versus controls using Graphpad Prism software. Values were expressed as mean ± SEM. A *p* value <0.05 was considered to indicate statistical significant difference and n refers to number of animals per group.

## Results

### Immune complexes in the brain parenchyma

**Fig. 1:**
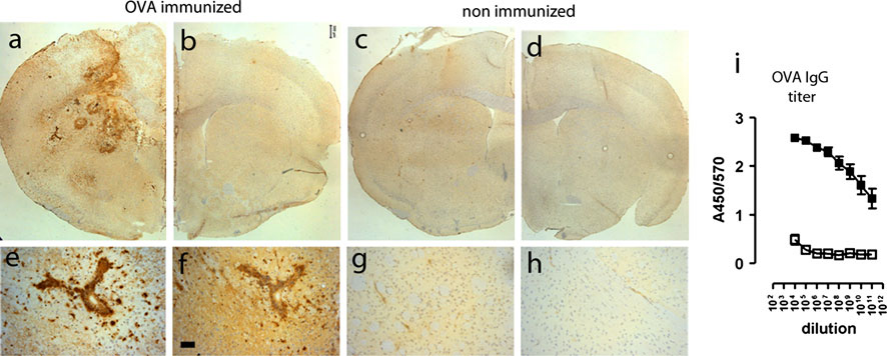
Accumulation of OVA and IgG following intracerebral injection of OVA. OVA-immunized mice (**a**, **b**, **e**, **f**) or non-immunized mice (**c**, **d**, **g**, **h**) received a unilateral injection of OVA into the striatum and were assessed for the presence of OVA and IgG 24 h later. Immunocytochemical analysis of OVA revealed accumulation of OVA at the injected site of the brains of immunized mice (**a**) and not in the injected site of the brain of non-immunized mice (**c**). The non-injected hemisphere of the brain of both immunized (**b**) and non-immunized mice (**d**) were negative for OVA immunoreactivity. *Scale bar* 300 μm. A higher magnification shows accumulation of OVA in association with and around blood vessels of OVA-immunized mice (**e**) but not in non-immunized mice (**g**). Immunocytochemical staining of IgG showed extravasation from blood vessels in OVA-immunized mice (**f**) but not in non-immunized mice (**h**). *Scale bar* 50 μm. Representative data of *n* = 3 per treatment group is shown. The experiment was performed twice independently with comparable results. **i** Levels of circulating anti-OVA antibodies (total IgG) were determined by ELISA. Data is expressed as A450/570 values. *Closed symbols* and represent OVA-immunized mice (*n* = 14) and open symbols represents non-immunized mice (*n* = 3)

**Fig. 2:**
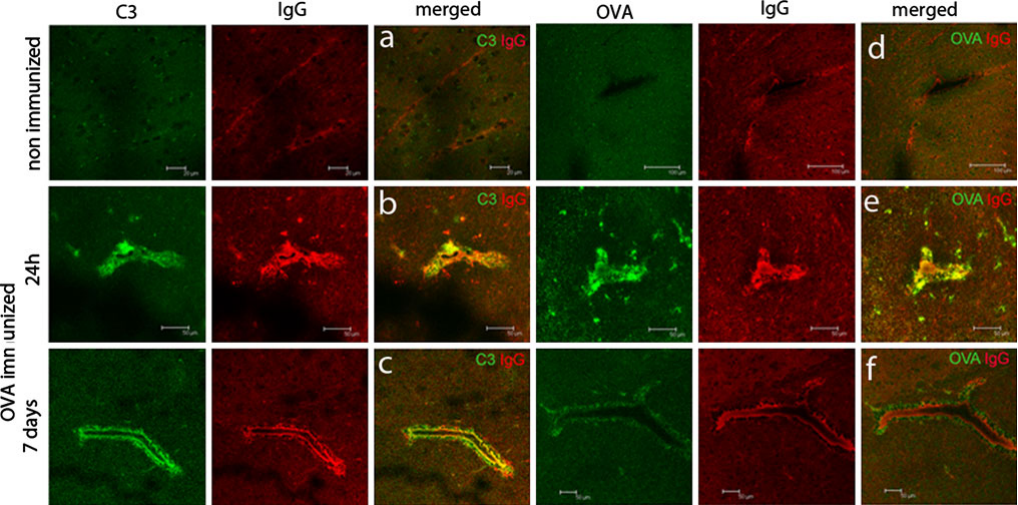
Immune complexes form in the parenchyma and in association with the vasculature after intracerebral injection of OVA. Non-immunized mice or OVA-immunized mice received a unilateral injection of OVA in the striatum and were assessed for presence of OVA, IgG and complement C3 at 24 h and 7 days. Data shows IgG in *red* co-stained for C3 or OVA in *green*. At 24 h OVA is present in the parenchyma and around blood vessels of immunized mice, while at later time points OVA is largely confined to the vicinity of the vasculature. Data shown is representative of *n* = 3 per treatment group. The experiment was performed twice independently with comparable results

Mice were sensitized to ovalbumin (OVA) followed by stereotaxic microinjection of OVA into the striatum to induce immune complex deposition in the brain parenchyma. Non-immunized mice received a similar intracerebral injection of OVA in the striatum and were used as controls. All immunized mice showed high levels of circulating OVA-specific antibodies, with IC50 values of >1:1,000,000 in our binding assay to OVA coated plates. Sera from non-immunized mice were negative (Fig. 1i). In OVA-sensitized mice, intracerebral injection of OVA resulted in the accumulation of OVA and IgG in the brain; the effect was restricted to the injected hemisphere of the brain although spread over the dorsal half (Fig. 1a, b). At 24 h after intracerebral injection of OVA, immunized mice showed accumulation of OVA (Fig. 1e) and IgG (Fig. 1f). Non-immunized mice that received a similar challenge with OVA did not show accumulation of these proteins (Fig. 1c–d, g–h). These results suggest that OVA-immunized mice have increased retention of antigen following intrastriatal challenge. To further investigate and characterize the immune complexes we analysed tissue sections for co-localization of OVA and IgG and complement component C3 by double immuno-fluorescent staining (Fig. 2). At 24 h after injection of OVA, we observed increased IgG immunoreactivity in the lumen of blood vessels and found IgG co-localized with C3 (Fig. 2b) and OVA (Fig. 2e) in the parenchyma. At 7 days after OVA injection, deposits of C3 and OVA immunoreactivity were still present in the brain of immunized mice, co-localizing with IgG around blood vessels (Fig. 2c, f). Immune complexes, as revealed by the co-localization of IgG with C3 and OVA, were observed not only close to the injection site, but also in association with blood vessels in the cortex, the leptomeninges and corpus callosum (data not shown). Non-immunized control mice did not show evidence of IgG or C3 co-localized with OVA in the brain (Fig. 2a, d). Together, these data suggest that immune complexes form in the parenchyma following intracerebral injection of OVA into sensitized mice, resulting in an accumulation of IgG and co-localization with C3 and OVA.

### Macrophage/microglial activation

**Fig. 3:**
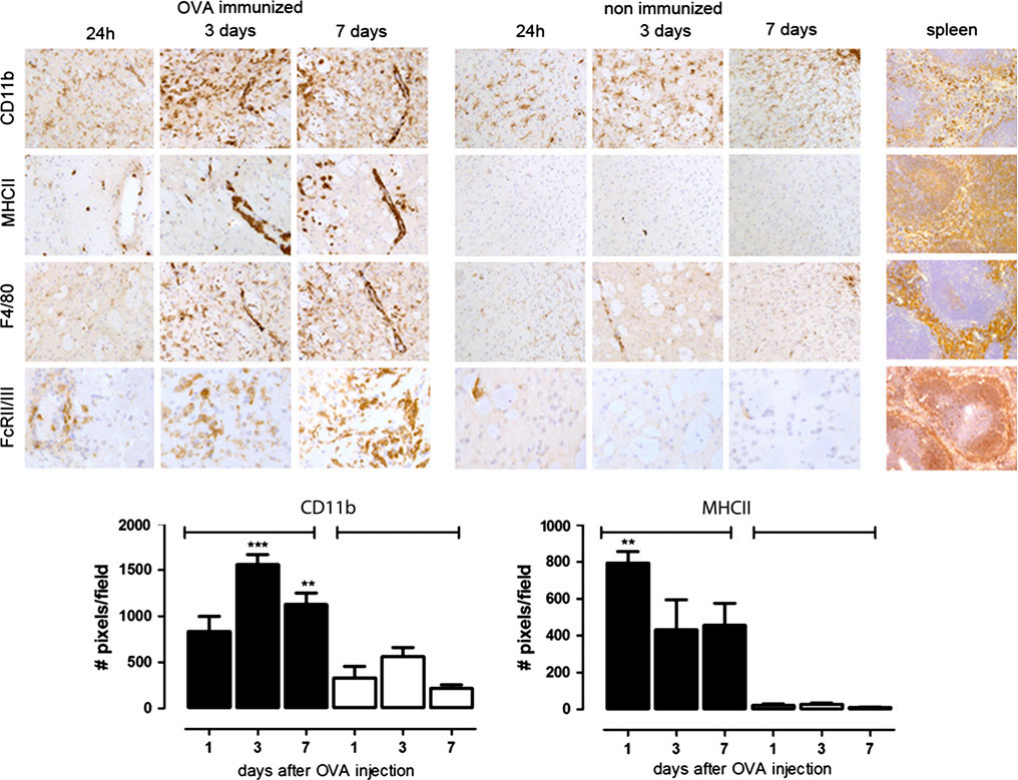
Macrophage and microglia activation in the brain after intracerebral injection of OVA. OVA-immunized mice or control non-immunized mice received a unilateral injection of OVA into the striatum and were assessed for presence of macrophage and microglia activation. Data shows CD11b, MHCII, F4/80 and FcγRII/III immunoreactivity after 24 h, 3 days or 7 days in OVA-immunized mice or non-immunized mice. As a positive control for these well-characterized antibodies spleen tissue from a naïve mouse was used. The number of DAB-positive pixels (cells and their processes) in OVA-immunized (*black bars*) and non-immunized mice (*white bars*) was quantified as described in “Materials and methods”. Statistical analysis: One-way ANOVA, Dunnett’s post-test *p < 0.05. Data represents the mean of *n* = 3–5 per treatment and time point. The experiment was performed twice independently with comparable results

The presence of OVA at the abluminal site of blood vessels suggests possible phagocytosis of the immune complexes by perivascular macrophages. Therefore, we next investigated whether macrophage and microglia activation could be observed in response to immune complexes. The myeloid markers CD11b and F4/80 are weakly expressed on microglia in the parenchyma of non-immunized mice and MHCII is undetectable. Immune complex formation in the brain was associated with increased expression of the CD11b, MHCII and F4/80. At 24 h after intracerebral injection of OVA, minor changes in expression were observed, but at 3 and 7 days after intracerebral challenge with OVA in immunized mice, perivascular macrophages and microglia changed morphology and phenotype, and showed markedly increased expression of CD11b, MHCII and F4/80 (Fig. 3). Quantification revealed that the increase in CD11b (*F*_(5,18)_ = 19.92, *p* < 0.0001) and MHC class II (*F*_(5,18)_ = 7.638, *p* = 0.0005) was significantly different from non-immunized mice that received a similar intracerebral injection of OVA (Fig. 3). We found that immune complex formation also induced increased expression of FcγII/III receptor (FcγR), which was already observed at 24 h after OVA injection. This increase in expression was first detectable on perivascular cells, followed by a clear expression on parenchymal cells, including microglia, at later time points (Fig. 3). At 7 days after OVA injection, a large number of macrophages and microglia showed increased expression of FcγRII/III, while non-immunized control mice showed minimal expression of FcγRs. These data suggest that immune complex formation induces microglia activation, possibly initiated by activation and increased expression of FcγRs on perivascular macrophages.

### Neuroinflammation in the absence of neutrophils

**Fig. 4:**
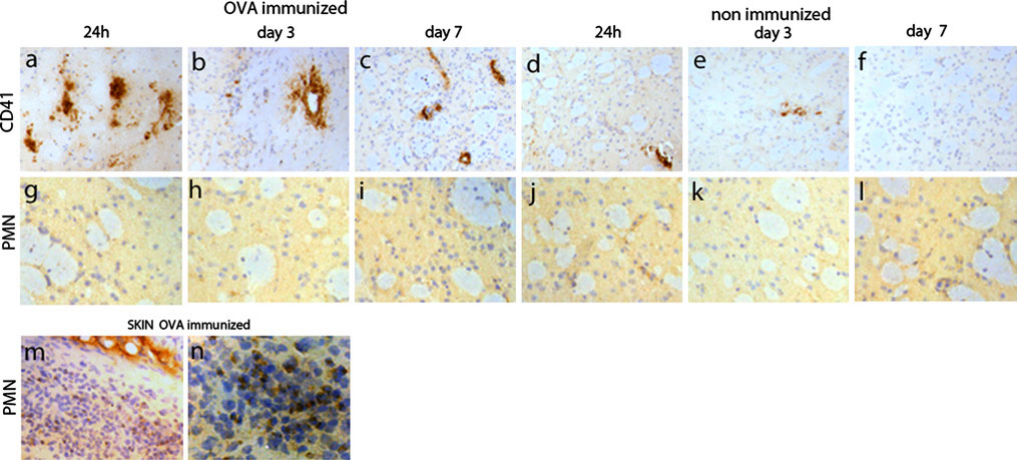
Platelet aggregation and neutrophil recruitment in the brain after intracerebral injection of OVA. OVA-immunized mice or control mice received a unilateral injection of OVA into the striatum and were assessed for presence of platelets (CD41) or polymorphonuclear cells (PMN). CD41 (**a**–**f**) or PMN (**g**–**l**) immunoreactivity is shown after 1 day, 3 days or 7 days in OVA-immunized mice or non-immunized mice. As a positive control, OVA was injected in the skin of an OVA-immunized mouse and assessed for PMN recruitment (**n**–**m**). Representative data of *n* = 3 per treatment and time point is shown. The experiment was performed once

To investigate if immune complexes in the brain induce leucocyte recruitment similar to that seen in peripheral tissues, we evaluated brain tissue for the presence of platelets and neutrophils at various time points after intracerebral injection of OVA. Immunocytochemical analysis revealed increased CD41 expression in OVA-immunized mice (Fig. 4), indicating the presence of platelets. Platelets were present mainly in and around blood vessels in the injected hemisphere from 24 h, persisting up to 7 days following OVA challenge (Fig. 4a–c). Minimal CD41 immunoreactivity was observed in non-immunized control mice (Fig. 4d–f). In contrast, neutrophils could not be detected in the brain perivascular space or parenchyma of either immunized or non-immunized mice (Fig. 4g–l). Injection of OVA into the skin of an OVA-immunized mouse confirmed recruitment of neutrophils at a peripheral site following immune complex formation (Fig. 4m, n).

**Fig. 5:**
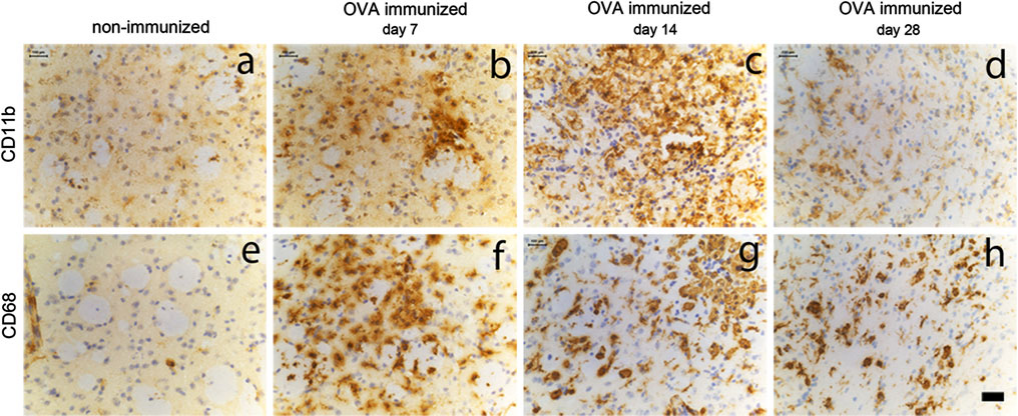
Kinetics of macrophage and microglia activation in the brain after intracerebral injection of OVA. OVA-immunized mice or non-immunized mice received a unilateral injection of OVA into the striatum and brain tissue was assessed for presence of macrophage activation at day 7, day 14 and day 28. Images in the top panel shows CD11b immuno-reactivity in control non-immunized mice (**a**) and after 7 days (**b**), 14 days (**c**) or 28 days (**d**) in OVA-immunized mice: CD68 immunoreactivity in the lower panel in control (**e**) and after 7 days (**f**), 14 days (**g**) and 28 days (**h**) in immunized mice. Representative data of *n* = 3 per treatment and time point is shown. *Scale bar* 50 μm

The long-term consequence of antibody-mediated neuroinflammation was assessed by staining for CD11b and CD68 expression at 7, 14 and 28 days after OVA injections with OVA. Figure 5 shows that expression of CD11b and CD68 was increased relative to non-immunized controls at 7 days after intracerebral OVA challenge (Fig. 5b, f), at 14 days (Fig. 5c, g) and even 28 days after OVA injection (Fig. 5d, h). Many of these cells had a rounded appearance indicative of both microglia activation and possible recruitment of monocytes across the damaged BBB.

### Differential role of complement and Fc receptors

**Fig. 6:**
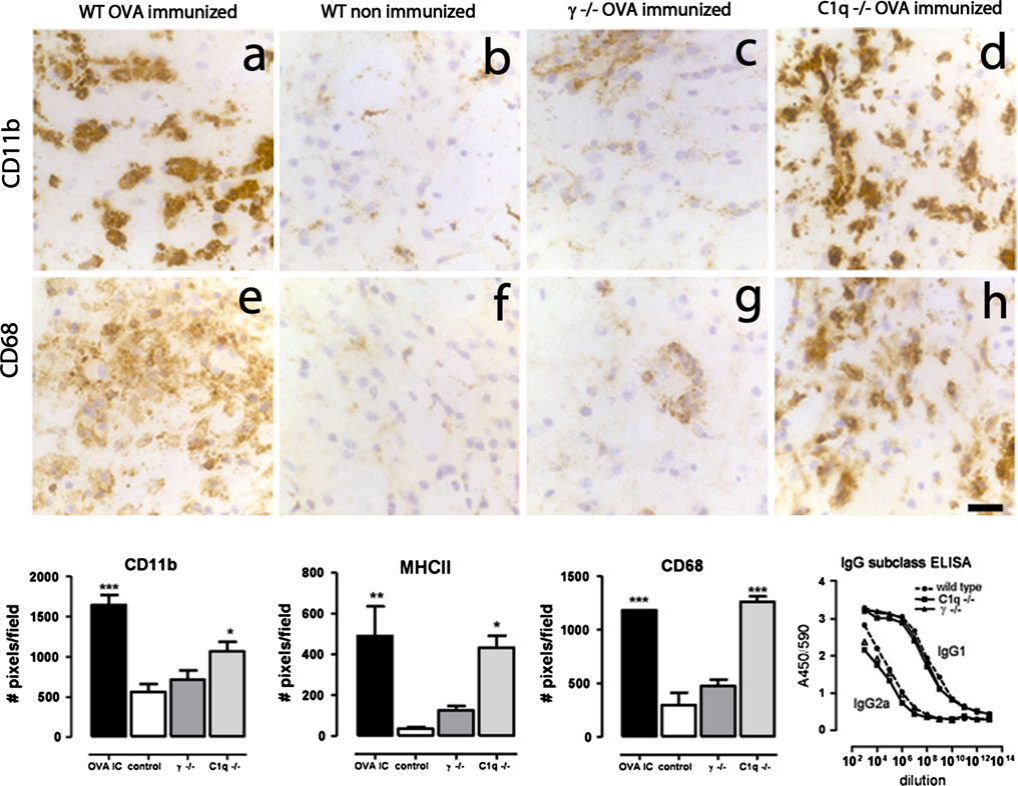
Macrophage and microglia activation in the brain after intracerebral injection of OVA in wild type, C1q−/− mice and Fcγ−/−. Wild type, C1q−/− mice and Fcγ−/− mice on a BALB/c background were immunized against OVA followed by unilateral injection of OVA into the striatum. Macrophage and microglia activation was assessed by immunocytochemistry. Images in the top panel show CD11b immunoreactivity 3 days after intracerebral injection of OVA in OVA-immunized wild type (**a**), non-immunized wild type (**b**) or OVA-immunized Fcγ−/− (**c**) and OVA-immunized C1q−/− (**d**). Images in the middle panel shows CD68 immunoreactivity 3 days after intracerebral injection of OVA in OVA-immunized wild type (**e**), non-immunized wild type (**f**), OVA-immunized Fcγ−/− (**g**) and OVA-immunized C1q−/− (**h**). *Scale bar* 50 μm. In the *bottom panel* the number of DAB-positive pixels/field (cells and their processes) in the injected hemisphere was quantified as described in “Materials and methods”. Levels of circulating anti-OVA antibodies (IgG1 and IgG2a) were determined by ELISA. Data is expressed as A450/570 values. *Closed circle* and *dotted line* represent wild type, *closed diamonds* represents C1q−/− mice and *closed squares* represents Fcγ−/− mice. Statistical analysis: One-way ANOVA, Dunnett’s post-test. Data are expressed as mean of *n* = 3–6 per group. The experiment was performed once

In order to understand the mechanisms underlying OVA–immune complex-mediated inflammatory changes in the brain parenchyma, we studied immune complex-mediated inflammatory responses in the striatum of C1q−/− and Fcγ chain−/− mice. Intracerebral injection of OVA in C1q−/− mice resulted in an increased expression of CD11b (Fig. 6d), CD68 (Fig. 6h) and FcγR (data not shown), similar to OVA-immunized wild-type controls (Fig. 6a, e). In contrast, Fcγ chain−/− showed only limited increase in expression of these macrophage markers (Fig. 6c, g). Comparison of macrophage activation between non-immunized wild type, OVA-immunized wild type, C1q−/− and Fcγ chain−/− mice revealed significant up-regulation of myeloid markers following OVA challenge [One-way ANOVA: CD11b (*F*_(3,15)_ = 19.70, *p* < 0.0001), CD68 (*F*_(3,11)_ = 46.68, *p* < 0.0001) and MHC class II (*F*_(3,19)_ = 6.496, p = 0.0044)]. Compared to non-immunized mice, a Dunnett’s post hoc analysis revealed that expression of CD11b, MHCII and CD68 was significantly different in OVA-immunized wild type and C1q−/− mice only, with no significant difference in OVA-immunized Fcγ chain−/− mice (Fig. 6). The levels of circulating anti-OVA antibodies were similar in wild-type, C1q−/− and FcγR−/− mice (Fig. 6). These results suggest that FcγRs, but not C1q, play an important role in the inflammatory response following antibody-mediated inflammation in the brain.

**Fig. 7:**
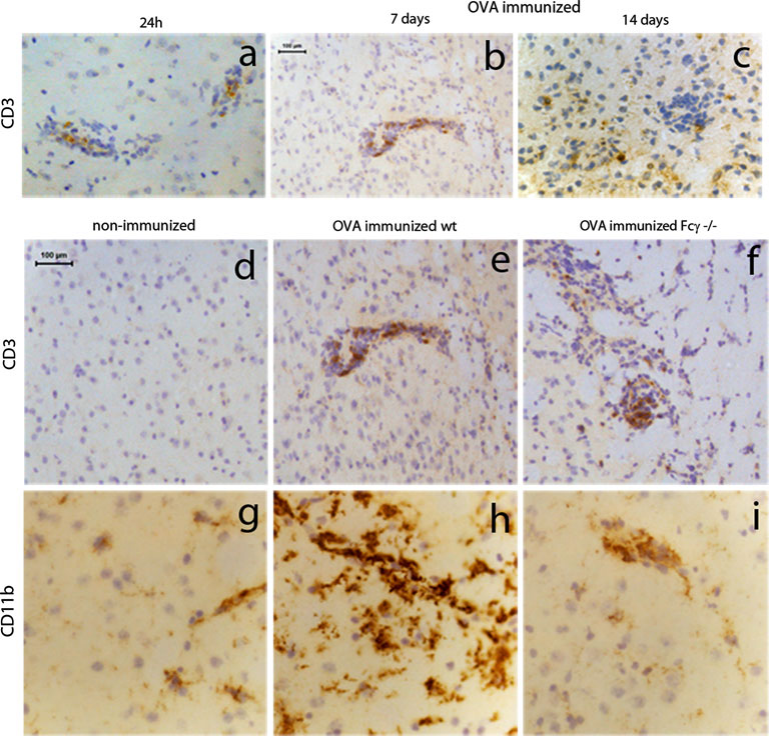
T-cell recruitment after immune complex formation. OVA-immunized mice received a unilateral injection of OVA into the striatum and brain tissue was assessed for presence of CD3-positive T cells at 24 h (**a**), day 7 (**b**) and day 14 (**c**) after OVA injection. Non-immunized mice were used as control (**d**, **g**). OVA-immunized wild type (**e**, **h**) and Fcγ−/− (**f**, **i**) mice received a unilateral injection of OVA into the striatum and tissue was analysed for CD3 (**d**, **e**, **f**) or CD11b (**g**, **h**, **i**) immunoreactivity at 7 days. Representative data of *n* = 2–3 per time point are shown. *Scale bar* 100 μm

To investigate if T cells are recruited to the brain following antibody-mediated neuroinflammation, we analysed brain sections for the presence of CD3 positive T cells at 24 h, 7 days and 14 days after intracerebral OVA injection. Small numbers of CD3+ T cells were detected in the vicinity of blood vessels at both 24 h and 7 days (Fig. 7a, b). A limited number of CD3+ T cells was detected in the brain parenchyma following immune complex formation 14 days earlier (Fig. 7c) while non-immunized control mice did not show immunoreactivity for CD3 (Fig. 7d). Next, we analysed the striatum of OVA-immunized γ−/− mice for the presence of T cells to compare with OVA-immunized wild-type mice. We found that both strains showed evidence of CD3+ T cells recruitment (Fig. 7e, f), but very different CD11b immunoreactivity (Fig. 7h, i) measured 7 days following intracerebral OVA injection.

### Behavioural changes

**Fig. 8:**
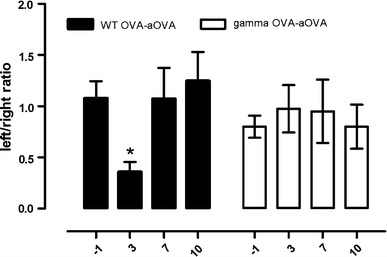
Behavioural changes after intracerebral injection of OVA in OVA-immunized wild-type or Fcγ−/− mice. The number of left and right turns was measured at baseline (−1 day) and then at day 3, day 7 and day 10 after intracerebral injection of OVA in OVA-immunized WT or Fcγ−/− mice. LPS was given intraperitoneally at a dose of 100 μg/kg and rotation behaviour was tested 3 h after injection. Data is presented as the ratio of left and right turns during a 1-min test. Statistical analysis: One-way ANOVA, Dunnett's post-test, *n* = 6 per time point, *p<0.05. The experiment was performed twice independently and results of experiments are pooled for the analysis

Rotation behaviour was used to investigate the functional consequences of antibody-mediated inflammation in the brain parenchyma. Altered rotation behaviour is an indication of neuronal damage to the striatum, which is elegantly shown by unilateral injections of the neurotoxin kainic acid [23]. We measured rotation behaviour over a 1 min period, at 0, 1, 3 or 7 days after intracerebral OVA injections in wild type and Fcγ−/− mice. There was no difference in the baseline ratio of left/right turns between OVA-immunized wild-type and OVA-immunized Fcγ−/− mice. Intracerebral OVA injection into the left striatum induced a significant decrease in the left:right ratio of OVA-immunized WT mice. A Dunnett’s post hoc test revealed that the alterations were significantly different from baseline levels when measured 3 days after OVA injection (*F*_(4,31)_ = 4.165, *p* = 0.0082, Fig. 8). Intracerebral OVA injection did not induce changes in rotation behaviour of OVA-immunized Fcγ−/− mice (*F*_(4,12)_ = 1.901, *p* = 0.1751), suggesting an important role for FcγRs in the functional consequences of immune complex formation in the brain (Fig. 8).

## Discussion

In this study we have demonstrated that the formation of immune complexes in the brain parenchyma results in a localized neuroinflammatory response and associated behavioural changes. We show that immune complexes form in association with cerebral blood vessels of OVA-sensitized mice that have received an intracerebral OVA challenge. Immune complex formation results in increased expression of FcγRII/III, CD11b, F4/80, CD68 and MHCII on perivascular macrophages and microglia, observed from 3 days until 4 weeks after antigen challenge. At 24 h we detected platelets adhering to blood vessels, but neutrophils were not detected at any time point measured. We further showed that the interaction with FcγRs is critical for the induction of this inflammatory response, since mice lacking the γ-chain did not show the histopathological changes or altered rotation behaviour, despite similar levels of circulating OVA-specific IgG. Some characteristics of immune complex-mediated inflammation in the CNS are similar to those observed in skin or lung inflammation models, including extravasation of IgG, activation of the complement cascade and activation of macrophages, but it differs significantly in the kinetics and cellular components recruited.

### The role of FcR in antibody-mediated neuroinflammation

The mechanisms of immune complex-mediated inflammation have been extensively studied in peripheral organs, such as the skin and the lung. It was shown that when antigen–antibody immune complexes are formed at vascular basement membranes in extracerebral sites, they trigger inflammation, characterized by oedema, recruitment of neutrophils, complement activation, and local tissue damage [10, 41]. It has been suggested that both the complement system and the activation of Fc receptors contribute to these inflammatory response [39, 40]. The effects of immune complex triggered inflammation in the brain and associated neurobehavioural consequences have only been sporadically reported in the literature. In 1932, Davidoff et al. reported that rabbits, sensitized against egg albumin showed a typical sterile inflammation characteristic for local anaphylactic symptoms, following intracerebral challenge with the same antigen. The pathological changes were similar to those described earlier in the skin [24], including tissue necrosis, oedema, haemorrhages and infiltration of leucocytes. The surviving rabbits developed behavioural changes, including tonic and clonic muscular contractions and rotating movements. These observations were the first to describe the devastating consequences of immune complex formation in the brain, but due to the relatively crude methodology and many fatalities, the results should be interpreted with care. Our study shows that there are important differences between immune complex-induced inflammation in the brain and other tissues, but highlight the similar role for FcγRs in the initiation of IgG-mediated inflammation and functional behavioural changes. Although components of complement appear less critical our study only used C1q−/− mice, and to rule out other factors of the complement system further studies are required. Previous studies using unilateral, intrastriatal injections of LPS have shown similar effects on microglia and circling behaviour [19]. The molecular mechanisms underlying these changes include increased expression of MHCII, cytokines and iNOS in the substantia nigra and striatum, but whether a similar mechanism explains the behavioural changes in our model remains undetermined.

A limited number of studies looked at the mechanism underlying neuropathology following immune complex formation, but the role of FcγR is largely unknown. Schupf and William [38] showed that injection of preformed OVA-anti-OVA immune complexes into the hypothalamus of rats results in increased food intake. As the effect was not observed upon injection of immune complexes containing F(ab′)_2_ fragments, the authors concluded that the effects observed depend on complement activation. However, as F(ab′)_2_ fragments lack Fc, interaction with FcγRs, cannot be excluded. A more recent study using a model of neuromyelitis optica (NMO) also suggests a key role for complement in the mechanism of antibody-mediated inflammation in the CNS [20]. Saadoun et al. [36] demonstrated that intracerebral injection of human IgG into mouse brain only induces pathology when co-injected with human complement. The pathology was characterized by infiltration of monocytes, but not granulocytes, and ipsiversive rotation behaviour, similar to our study. FcγRs display highest affinity to IgG of the same species [1] possibly explaining why human antibody alone did not induce pathology, while mouse antibodies in our study do so.

### Initiation of antibody-mediated neuroinflammation

Immunohistochemical data show that immune complexes deposit in the brain parenchyma within 24 h after antigen challenge. We cannot rule out that the use of a micropipette for intracerebral OVA challenge induces BBB leakage. However, immune complexes were not observed solely in the injection site, but observed throughout the challenged hemisphere. In addition, intra-vitreous injections that do not damage the blood–retinal barrier (BRB) lead to a similar inflammatory response (unpublished observations), suggesting an alternative mechanism for increased IgG influx into the CNS. It is generally believed that circulating antibodies are restricted to enter the brain due to an intact BBB, but it has been shown that very low levels of IgG (~0.1 %) can gain access to the brain via the extracellular pathway [5]. We show that mice used in our study have high serum titers of OVA-specific antibodies, and under these conditions, the low level of IgG that crosses the intact BBB is possibly sufficient to initiate the deposition of immune complexes. The perivascular space is likely the primary site of immune complex-induced inflammation as we find constitutive and then rapidly increased expression of FcγR on perivascular macrophages. It has also been suggested that activated T cells play a role the breaking the integrity of the BBB in models of auto-immune-mediated neurological diseases. Hu et al. [18] show that in the presence of antigen, activated T cells extravasate into ocular tissue, resulting in a monocyte recruitment and further breakdown of the BRB. Similar findings have been described in animal models of demyelination in the spinal cord [44], but high numbers (3–5 × 10^6^) of T cells were needed to induce opening of the BBB. We detected small numbers of CD3+ T cells in our model, therefore, we cannot exclude a role for T cells in altering BBB permeability and increased IgG influx. However, CD3+ T cells were also observed in FcγR−/−, suggesting that increased microglial activation depends on interaction with FcγRs.

### Antibody-mediated neuropathology

In the present study we show that OVA-anti-OVA immune complexes-induced expression of CD11b, CD68, MHC class II as well as marked expression levels of FcγRs on microglia. Similar results have been reported following intracerebral injection of anti-Aβ antibodies in APP transgenic mice [49]. Humoral components have been implicated in the pathogenesis of neurodegenerative diseases although it is controversial as to what extent the antibodies are pathogenic or simply a consequence of the ongoing neurodegeneration [11]. Engelhardt et al. [13] showed loss of cholinergic neurons following injection of IgG isolated from an AD patient. Similar observations were made using IgG derived from PD patients resulting in microglial activation and loss of hydroxylase (TH+) neurons. Intracerebral administration of PD-derived IgG results in perivascular inflammation, significant microglial activation and increased rotational behaviour [9]. Interestingly, the effects on microglial activation were absent in FcγR−/− mice [16]. Postmortem analysis of PD brain tissue shows similar histopathological changes as those observed in the animal models [22]. These observations suggest that, apart from classic CNS autoimmune disorders, immune complexes may contribute to the pathogenesis and/or ongoing pathology of neurodegenerative diseases.

### Implications for immunotherapy

There is growing academic and commercial interest in utilizing the power of antibodies to treat AD by vaccination against Aβ peptides [37] but immunotherapy targeting of brain antigens is not without risks. Histological examination of the brains of immunized humans reveals that, although immunization reduces the plaque load in the parenchyma [29], vascular Aβ deposits persist, leading to increased incidence of haemorrhages [6]. Experimental models have shown that antibodies devoid of the Fc region, such as Fab′, F(ab′)2 and scFv antibodies, or de-glycosylated antibodies, which cannot engage effector systems, successfully remove Aβ from the brain without inducing haemorrhages [14, 33, 45]. Furthermore, increased expression of FcγR expression levels are reported following passive immunization with Aβ-specific antibodies in APP transgenic mice, which did not occur after deglycosylation of the therapeutic antibody [8, 48]. These observations suggest that antibodies facilitate in the removal of plaques, but their Fc regions can cause detrimental inflammatory reactions through interaction with perivascular macrophages and microglia. Another potential side-effect of immunotherapy is the solubilization of Aβ peptides from plaques that remain trapped in the perivascular drainage pathways, leading to worsening of cerebral amyloid angiopathy [6]. We hypothesize that formation of immune complexes between Aβ peptides and Aβ antibodies and subsequent inflammation may partly explain increased CAA following immunotherapy. A better understanding of FcγRs and controlling FcγR function in the brain microenvironment will likely increase the success of immunotherapy for neurodegenerative diseases and reduce clinical setbacks experienced to date.

## References

[1] Alexander EL, Sanders SK (1977). F(ab’)2 reagents are not required if goat, rather than rabbit, antibodies are used to detect human surface immunoglobulin. J Immunol.

[2] Andersson PB, Perry VH, Gordon S (1992). The acute inflammatory response to lipopolysaccharide in CNS parenchyma differs from that in other body tissues. Neuroscience.

[3] Andersson PB, Perry VH, Gordon S (1992). Intracerebral injection of proinflammatory cytokines or leukocyte chemotaxins induces minimal myelomonocytic cell recruitment to the parenchyma of the central nervous system. J Exp Med.

[4] Banks WA, Erickson MA (2010). The blood–brain barrier and immune function and dysfunction. Neurobiol Dis.

[5] Banks WA, Terrell B, Farr SA, Robinson SM, Nonaka N, Morley JE (2002). Passage of amyloid beta protein antibody across the blood–brain barrier in a mouse model of Alzheimer’s disease. Peptides.

[6] Boche D, Zotova E, Weller RO, Love S, Neal JW, Pickering RM, Wilkinson D, Holmes C, Nicoll JA (2008). Consequence of Abeta immunization on the vasculature of human Alzheimer’s disease brain. Brain.

[7] Botto M, Dell’Agnola C, Bygrave AE, Thompson EM, Cook HT, Petry F, Loos M, Pandolfi PP, Walport MJ (1998). Homozygous C1q deficiency causes glomerulonephritis associated with multiple apoptotic bodies. Nat Genet.

[8] Carty NC, Wilcock DM, Rosenthal A, Grimm J, Pons J, Ronan V, Gottschall PE, Gordon MN, Morgan D (2006). Intracranial administration of deglycosylated C-terminal-specific anti-Abeta antibody efficiently clears amyloid plaques without activating microglia in amyloid-depositing transgenic mice. J Neuroinflammation.

[9] Chen S, Le WD, Xie WJ, Alexianu ME, Engelhardt JI, Siklos L, Appel SH (1998). Experimental destruction of substantia nigra initiated by Parkinson disease immunoglobulins. Arch Neurol.

[10] Crawford JP, Movat HZ, Minta JO, Opas M (1985). Acute inflammation induced by immune complexes in the microcirculation. Exp Mol Pathol.

[11] D’Andrea MR (2003). Evidence linking neuronal cell death to autoimmunity in Alzheimer’s disease. Brain Res.

[12] Davidoff LM, Seegal BC, Seegal D (1932). The arthus phenomenon: local anaphylactic inflammation in the rabbit brain. J Exp Med.

[13] Engelhardt JI, Le WD, Siklos L, Obal I, Boda K, Appel SH (2000). Stereotaxic injection of IgG from patients with Alzheimer disease initiates injury of cholinergic neurons of the basal forebrain. Arch Neurol.

[14] Fukuchi K, Tahara K, Kim HD, Maxwell JA, Lewis TL, Accavitti-Loper MA, Kim H, Ponnazhagan S, Lalonde R (2006). Anti-Abeta single-chain antibody delivery via adeno-associated virus for treatment of Alzheimer’s disease. Neurobiol Dis.

[15] Galea I, Bechmann I, Perry VH (2007). What is immune privilege (not)?. Trends Immunol.

[16] He Y, Le WD, Appel SH (2002). Role of Fcgamma receptors in nigral cell injury induced by Parkinson disease immunoglobulin injection into mouse substantia nigra. Exp Neurol.

[17] Herber DL, Maloney JL, Roth LM, Freeman MJ, Morgan D, Gordon MN (2006). Diverse microglial responses after intrahippocampal administration of lipopolysaccharide. Glia.

[18] Hu P, Pollard JD, Chan-Ling T (2000). Breakdown of the blood–retinal barrier induced by activated T cells of nonneural specificity. Am J Pathol.

[19] Hunter RL, Cheng B, Choi DY, Liu M, Liu S, Cass WA, Bing G (2009). Intrastriatal lipopolysaccharide injection induces parkinsonism in C57/B6 mice. J Neurosci Res.

[20] Jarius S, Aboul-Enein F, Waters P, Kuenz B, Hauser A, Berger T, Lang W, Reindl M, Vincent A, Kristoferitsch W (2008). Antibody to aquaporin-4 in the long-term course of neuromyelitis optica. Brain.

[21] Lambert JC, Heath S, Even G, Campion D, Sleegers K, Hiltunen M, Combarros O, Zelenika D, Bullido MJ, Tavernier B, Letenneur L, Bettens K, Berr C, Pasquier F, Fievet N, Barberger-Gateau P, Engelborghs S, De Deyn P, Mateo I, Franck A, Helisalmi S, Porcellini E, Hanon O, de Pancorbo MM, Lendon C, Dufouil C, Jaillard C, Leveillard T, Alvarez V, Bosco P, Mancuso M, Panza F, Nacmias B, Bossu P, Piccardi P, Annoni G, Seripa D, Galimberti D, Hannequin D, Licastro F, Soininen H, Ritchie K, Blanche H, Dartigues JF, Tzourio C, Gut I, Van Broeckhoven C, Alperovitch A, Lathrop M, Amouyel P (2009). Genome-wide association study identifies variants at CLU and CR1 associated with Alzheimer’s disease. Nat Genet.

[22] Le W, Rowe D, Xie W, Ortiz I, He Y, Appel SH (2001). Microglial activation and dopaminergic cell injury: an in vitro model relevant to Parkinson’s disease. J Neurosci.

[23] Leigh PN, Reavill C, Jenner P, Marsden CD (1983). Basal ganglia outflow pathways and circling behaviour in the rat. J Neural Transm.

[24] Lewis PA (1908). The induced susceptibility of the guinea-pig to the toxic action of the blood serum of the horse. J Exp Med.

[25] Lister KJ, Hickey MJ (2006). Immune complexes alter cerebral microvessel permeability: roles of complement and leukocyte adhesion. Am J Physiol Heart Circ Physiol.

[26] Loughlin AJ, Woodroofe MN, Cuzner ML (1992). Regulation of Fc receptor and major histocompatibility complex antigen expression on isolated rat microglia by tumour necrosis factor, interleukin-1 and lipopolysaccharide: effects on interferon-gamma induced activation. Immunology.

[27] Lunnon K, Teeling JL, Tutt AL, Cragg MS, Glennie MJ, Perry VH (2011). Systemic inflammation modulates Fc receptor expression on microglia during chronic neurodegeneration. J Immunol.

[28] Morohoshi K, Goodwin AM, Ohbayashi M, Ono SJ (2009). Autoimmunity in retinal degeneration: autoimmune retinopathy and age-related macular degeneration. J Autoimmun.

[29] Nicoll JA, Wilkinson D, Holmes C, Steart P, Markham H, Weller RO (2003). Neuropathology of human Alzheimer disease after immunization with amyloid-beta peptide: a case report. Nat Med.

[30] Perry VH, Andersson PB (1992). The inflammatory response in the CNS. Neuropathol Appl Neurobiol.

[31] Perry VH, Hume DA, Gordon S (1985). Immunohistochemical localization of macrophages and microglia in the adult and developing mouse brain. Neuroscience.

[32] Poduslo JF, Curran GL, Berg CT (1994). Macromolecular permeability across the blood-nerve and blood–brain barriers. Proc Natl Acad Sci USA.

[33] Poduslo JF, Ramakrishnan M, Holasek SS, Ramirez-Alvarado M, Kandimalla KK, Gilles EJ, Curran GL, Wengenack TM (2007). In vivo targeting of antibody fragments to the nervous system for Alzheimer’s disease immunotherapy and molecular imaging of amyloid plaques. J Neurochem.

[34] Ravetch JV (2002). A full complement of receptors in immune complex diseases. J Clin Invest.

[35] Ryu E, Fridley BL, Tosakulwong N, Bailey KR, Edwards AO (2010). Genome-wide association analyses of genetic, phenotypic, and environmental risks in the age-related eye disease study. Molecular vision.

[36] Saadoun S, Waters P, Bell BA, Vincent A, Verkman AS, Papadopoulos MC (2010). Intra-cerebral injection of neuromyelitis optica immunoglobulin G and human complement produces neuromyelitis optica lesions in mice. Brain.

[37] Schenk D, Barbour R, Dunn W, Gordon G, Grajeda H, Guido T, Hu K, Huang J, Johnson-Wood K, Khan K, Kholodenko D, Lee M, Liao Z, Lieberburg I, Motter R, Mutter L, Soriano F, Shopp G, Vasquez N, Vandevert C, Walker S, Wogulis M, Yednock T, Games D, Seubert P (1999). Immunization with amyloid-beta attenuates Alzheimer-disease-like pathology in the PDAPP mouse. Nature.

[38] Schupf N, Williams CA (1987). Complement-dependence of immune complex activity in the rat hypothalamus. Ann N Y Acad Sci.

[39] Shushakova N, Skokowa J, Schulman J, Baumann U, Zwirner J, Schmidt RE, Gessner JE (2002). C5a anaphylatoxin is a major regulator of activating versus inhibitory FcgammaRs in immune complex-induced lung disease. J Clin Invest.

[40] Sylvestre DL, Ravetch JV (1994). Fc receptors initiate the Arthus reaction: redefining the inflammatory cascade. Science.

[41] Szalai AJ, Digerness SB, Agrawal A, Kearney JF, Bucy RP, Niwas S, Kilpatrick JM, Babu YS, Volanakis JE (2000). The Arthus reaction in rodents: species-specific requirement of complement. J Immunol.

[42] Takai T, Li M, Sylvestre D, Clynes R, Ravetch JV (1994). FcR gamma chain deletion results in pleiotrophic effector cell defects. Cell.

[43] Teeling JL, Felton LM, Deacon RM, Cunningham C, Rawlins JN, Perry VH (2007). Sub-pyrogenic systemic inflammation impacts on brain and behavior, independent of cytokines. Brain Behav Immun.

[44] Westland KW, Pollard JD, Sander S, Bonner JG, Linington C, McLeod JG (1999). Activated non-neural specific T cells open the blood–brain barrier to circulating antibodies. Brain.

[45] Wilcock DM, Alamed J, Gottschall PE, Grimm J, Rosenthal A, Pons J, Ronan V, Symmonds K, Gordon MN, Morgan D (2006). Deglycosylated anti-amyloid-beta antibodies eliminate cognitive deficits and reduce parenchymal amyloid with minimal vascular consequences in aged amyloid precursor protein transgenic mice. J Neurosci.

[46] Wilcock DM, Colton CA (2008). Anti-amyloid-beta immunotherapy in Alzheimer’s disease: relevance of transgenic mouse studies to clinical trials. J Alzheimers Dis.

[47] Wilcock DM, Colton CA (2009). Immunotherapy, vascular pathology, and microhemorrhages in transgenic mice. CNS Neurol Disord Drug Targets.

[48] Wilcock DM, Rojiani A, Rosenthal A, Levkowitz G, Subbarao S, Alamed J, Wilson D, Wilson N, Freeman MJ, Gordon MN, Morgan D (2004). Passive amyloid immunotherapy clears amyloid and transiently activates microglia in a transgenic mouse model of amyloid deposition. J Neurosci.

[49] Wilcock DM, Rojiani A, Rosenthal A, Subbarao S, Freeman MJ, Gordon MN, Morgan D (2004). Passive immunotherapy against Abeta in aged APP-transgenic mice reverses cognitive deficits and depletes parenchymal amyloid deposits in spite of increased vascular amyloid and microhemorrhage. J Neuroinflammation.

